# Monitoring Water Sources for Environmental Reservoirs of Toxigenic *Vibrio cholerae* O1, Haiti

**DOI:** 10.3201/eid2101.140627

**Published:** 2015-01

**Authors:** Stanislas Rebaudet, Renaud Piarroux

**Affiliations:** Aix-Marseille Université, Marseille, France

**Keywords:** *Vibrio cholerae*, O1, non-O1, 01, non-01, Haiti, environmental reservoirs, bacteria, waterborne, cholera, toxigenic

**To the Editor:** In the March 2014 issue of Emerging Infectious Diseases, Alam et al. reported a survey of water sources in Haiti conducted to isolate *Vibrio cholerae* (*1*). Each month from April 2012 through March 2013, they sampled 15 sites at 3 rivers and 1 estuary in West Department. From 179 water samples and 144 aquatic animals and plants, they obtained 7 *V. cholerae* O1 isolates, including 3 *ctx*-positive toxigenic strains. 

Unfortunately, the results for all 7 *V. cholerae* O1 isolates were aggregated, and no details were provided about the exact time and location of collection of samples corresponding to the 3 *ctx*-positive strains. The authors posed the question of whether *V. cholerae* O1 has become established in environmental reservoirs in Haiti, subsequently warning that “as long as the causative microorganism is present in the environment, eradication of the disease will not be possible.”

However, after challenging their results with more accurate epidemiologic data, we found that these 3 *ctx*-positive toxigenic strains could more likely have been present in the sampled rivers as a result of recent fecal contamination (Figure). Indeed, many cholera cases were reported in the corresponding communal sections (i.e., the smallest Haitian administrative unit, average 25 km^2^) when the samples containing the 7 *V. cholerae* O1 isolates were collected. In this context of an ongoing cholera epidemic associated with persisting rainfall (Figure), generalized open-air defecation inevitably leads to contamination of water sources. It is therefore impossible to determine whether *V. cholerae*–positive rivers constitute perennial reservoirs of the bacteria or whether they act only as transient vectors of the pathogens. 

**Figure:**
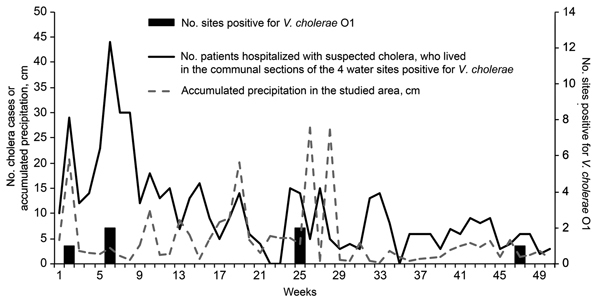
Weekly cholera incidence in communal sections of Haiti in which water samples were positive for *Vibrio cholerae* O1; accumulated precipitation in the studied area by week during April 2012–March 2013; and number of environmental sites from which *V. cholerae* O1 was isolated, by month. Incidence was calculated from patients who were hospitalized in the Leogane cholera treatment center and who resided near the 4 sites found positive for *V. cholerae* O1 by Alam et al. (*1*): second communal section of Leogane for Lassale site; second communal section of Gressier for Gressier and Gressier Beach sites; and third communal section of Gressier for Jeffra site. Satellite-measured rainfall for 18.5°–18.6°N, 72.6°–72.5°W was extracted from http://disc2.nascom.nasa.gov/Giovanni/tovas/realtime.3B42RT_daily.2.shtml.

The recent dramatic decrease in cholera transmission may provide a good opportunity to address this issue (*2*). We thus encourage Alam et al. to continue the search for *ctx*-positive toxigenic *V. cholerae* O1 strains in surface waters, especially during cholera-free periods.
